# Supplementary material for: Genome-wide association and Mendelian randomisation analysis provide insights into the pathogenesis of heart failure
Source: Nat Commun. 2020 Jan 9;11:163. doi: 10.1038/s41467-019-13690-5 (PMC6952380; doi:10.1038/s41467-019-13690-5)
Supplement: Supplementary file 3 — Description of Additional Supplementary Files [file 41467_2019_13690_MOESM3_ESM.pdf]

Title: Supplementary Data 1
Description: Previously reported loci for heart failure and dilated cardiomyopathy

Title: Supplementary Data 2
Description: Association of sentinel variants or proxies with diseases and traits in NHGRI-EBI GWAS catalog

Title: SupplementaryData 3
Description: Association of sentinel variants with diseases and traits in UK Biobank

Title: Supplementary Data 4
Description: Genome-wide significant associations of sentinel variants or proxies with heart failure related traits

Title: Supplementary Data 5
Description: Associations of sentinel variants with heart failure after conditioning on risk factor traits using mtCOJO

Title: Supplementary Data 6
Description: MAGMA gene-based association analysis

Title: Supplementary Data 7
Description: Sentinel variants or proxies with missense protein coding consequence

Title: Supplementary Data 8
Description: Significant eQTL associations for sentinel HF variants in heart tissue

Title: Supplementary Data 9
Description: Significant protein quantitative trait locus (pQTL) associations for sentinel HF variants in whole blood

Title: Supplementary Data 10
Description: Mendelian randomisation analysis of the effect of risk factor traits on HF risk

Title: Supplementary Data 11
Description: Participating study definitions for heart failure

Title: Supplementary Data 12
Description: Characteristics of participating studies

Title: Supplementary Data 13
Description: Summary of genotyping, QC, imputation and analysis per study
