# Supplementary material for: Genome-wide association and Mendelian randomisation analysis provide insights into the pathogenesis of heart failure
Source: Nat Commun. 2020 Jan 9;11:163. doi: 10.1038/s41467-019-13690-5 (PMC6952380; doi:10.1038/s41467-019-13690-5)
Supplement: Supplementary file 17 — Reporting Summary [file 41467_2019_13690_MOESM17_ESM.pdf]

nature research

Corresponding author(s): R Thomas Lumbers

Last updated by author(s): Nov 8, 2019

Reporting Summary

Nature Research wishes to improve the reproducibility of the work that we publish. This form provides structure for consistency and transparency in reporting. For further information on Nature Research policies, see Authors & Referees and the Editorial Policy Checklist.

Statistics

For all statistical analyses, confirm that the following items are present in the figure legend, table legend, main text, or Methods section.

n/a	Confirmed	
☐	☒	The exact sample size (*n*) for each experimental group/condition, given as a discrete number and unit of measurement
☐	☒	A statement on whether measurements were taken from distinct samples or whether the same sample was measured repeatedly
☐	☒	The statistical test(s) used AND whether they are one- or two-sided<br>*Only common tests should be described solely by name; describe more complex techniques in the Methods section.*
☐	☒	A description of all covariates tested
☐	☒	A description of any assumptions or corrections, such as tests of normality and adjustment for multiple comparisons
☐	☒	A full description of the statistical parameters including central tendency (e.g. means) or other basic estimates (e.g. regression coefficient) AND variation (e.g. standard deviation) or associated estimates of uncertainty (e.g. confidence intervals)
☐	☒	For null hypothesis testing, the test statistic (e.g. *F*, *t*, *r*) with confidence intervals, effect sizes, degrees of freedom and *P* value noted<br>*Give P values as exact values whenever suitable.*
☐	☒	For Bayesian analysis, information on the choice of priors and Markov chain Monte Carlo settings
☒	☐	For hierarchical and complex designs, identification of the appropriate level for tests and full reporting of outcomes
☐	☒	Estimates of effect sizes (e.g. Cohen's *d*, Pearson's *r*), indicating how they were calculated

Our web collection on statistics for biologists contains articles on many of the points above.

Software and code

Policy information about availability of computer code

Data collection: NA

Data analysis:
CADD as implemented in FUMAv1.3.2 https://cadd.gs.washington.edu/
Eagle 2.3 https://data.broadinstitute.org/alkesgroup/Eagle/
EasyQC 9.2 https://www.uni-regensburg.de/medizin/epidemiologie-praeventivmedizin/genetische-epidemiologie/software/
FUMA 1.3.2 http://fuma.ctglab.nl/
GCTA 1.91.7beta https://cnsgenomics.com/software/gcta/
GSMR as implementes in GCTA1.91.7beta https://cnsgenomics.com/software/gcta/#GSMR
HaploReg 4.1 https://pubs.broadinstitute.org/mammals/haploreg/haploreg.php
IMPUTE2 v2, v3 http://mathgen.stats.ox.ac.uk/impute/impute_v2.html
LDSC 1.0.0 https://github.com/bulik/ldsc
MaCH 1.0.15 http://csg.sph.umich.edu/abecasis/mach/index.html
mach2dat 1 https://genome.sph.umich.edu/wiki/Mach2dat:_Association_with_MACH_output
MAGMA as implemented in FUMAv1.3.2 https://ctg.cncr.nl/software/magma
METAL 5-Mar-11 https://genome.sph.umich.edu/wiki/METAL
MetaXScan 0.6.1 https://github.com/hakyimlab/MetaXcan
Michigan Imputation Server v1.0.1 https://imputationserver.sph.umich.edu
minimac v3 https://genome.sph.umich.edu/wiki/Minimac
PLINK2 v2 https://www.cog-genomics.org/plink/2.0/
ProbABEL v0.5.0 https://github.com/GenABEL-Project/ProbABEL
QuickTest 1.1 https://wp.unil.ch/sgg/quicktest/
R v3.5.2 https://cran.r-project.org/
SHAPEIT v2, v3 https://mathgen.stats.ox.ac.uk/genetics_software/shapeit/shapeit.html
SNPTEST v2.5 https://mathgen.stats.ox.ac.uk/genetics_software/snptest/snptest.html
TwoSampleMR 0.4.15 https://github.com/MRCIEU/TwoSampleMR
Coloc v3.1 https://cran.r-project.org/web/packages/coloc/index.html

For manuscripts utilizing custom algorithms or software that are central to the research but not yet described in published literature, software must be made available to editors/reviewers. We strongly encourage code deposition in a community repository (e.g. GitHub). See the Nature Research guidelines for submitting code & software for further information.

Data

Policy information about availability of data

All manuscripts must include a data availability statement. This statement should provide the following information, where applicable:
- Accession codes, unique identifiers, or web links for publicly available datasets
- A list of figures that have associated raw data
- A description of any restrictions on data availability

Data availability. The data sets generated during this study are available from the corresponding author upon reasonable request. The summary estimates for this analysis are available on the Cardiovascular Disease Knowledge Portal (http://www.broadcvdi.org/).

Field-specific reporting

Please select the one below that is the best fit for your research. If you are not sure, read the appropriate sections before making your selection.

☒ Life sciences ☐ Behavioural & social sciences ☐ Ecological, evolutionary & environmental sciences

For a reference copy of the document with all sections, see nature.com/documents/nr-reporting-summary-flat.pdf

Life sciences study design

All studies must disclose on these points even when the disclosure is negative.

Sample size: Participants from 26 cohorts (with a total of 29 distinct datasets) with either a case-control or population-based study design were included in the meta-analysis, as part of the Heart Failure Molecular Epidemiology for Therapeutic Targets (HERMES) Consortium. Data from a total of 47,309 cases and 930,014 controls were analysed.

Data exclusions: Only samples of European ancestry were included. Samples and variants were excluded at both pre-impuation and post-imputation stages. Pre-imputation exclusion were study-specific and consisted of exclusions based on sample call rate, heterozygosity outliers, ancestry outliers, related individuals and sex mismatches, variant call rate, deviations from Hardy-Weinberg, high discordance rates, allele frequency. Post-imputation and prior to meta-analysis, variants were excluded if they satisfied any one of the following criteria: imputation quality < 0.5, minor allele frequency < 0.01, absolute betas and standard errors > 10. After meta-analysis, variants not present in more than 50% of studies were excluded.

Replication: Given the sample size and unavailability of a replication sample of sufficient size, experimental replication was not attempted.

Randomization: Cases were participants with diagnosed with HF. Controls were participants free of heart failure.

Blinding: Blinding was not relevant to our study. Only summary level data was shared by each participating study for meta-analysis.

Reporting for specific materials, systems and methods

We require information from authors about some types of materials, experimental systems and methods used in many studies. Here, indicate whether each material, system or method listed is relevant to your study. If you are not sure if a list item applies to your research, read the appropriate section before selecting a response.

Materials & experimental systems

n/a	Involved in the study	
☒	☐	Antibodies
☒	☐	Eukaryotic cell lines
☒	☐	Palaeontology
☒	☐	Animals and other organisms
☐	☒	Human research participants
☒	☐	Clinical data

Methods

n/a	Involved in the study	
☒	☐	ChIP-seq
☒	☐	Flow cytometry
☒	☐	MRI-based neuroimaging

Human research participants

Policy information about studies involving human research participants

Population characteristics: Participants were of European ancestry. Detailed population characteristics for each study are provided in Supplementary Data 12 and Supplementary Information.

Recruitment: Study participants were from either case-control or population-based study designs

Ethics oversight: All included studies were ethically approved by local institutional review boards and all participants provided written informed consent.

Note that full information on the approval of the study protocol must also be provided in the manuscript.
